# Molecular docking analysis of fluoroquinolones and other natural and synthetic compounds with the HCV NS3 helicase

**DOI:** 10.6026/97320630018147

**Published:** 2022-03-31

**Authors:** Ahtesham Ul Haq, Alisalman Sheikh, Sadaf Naeem, Syed Hani Abidi

**Affiliations:** 1 Department of Biochemistry, University of Karachi, Karachi-Pakistan; 2 Department of Biological and Biomedical Sciences, Aga Khan University, Karachi-Pakistan

**Keywords:** HCV, NS3 helicase, antiviral activity, fluoroquinolones

## Abstract

It is of an interest to document the molecular docking analysis of fluoroquinolones and other natural and synthetic compounds with the HCV NS3 helicase. Data shows that three fluoroquinolones interacted with the NS3 helicase in the catalytic region, targeting some of the amino acids known to play a crucial role in NS3 helicase activity. Similarly, binding energy shows that the fluoroquinolones were comparable to the thiazolpiperazinyl derivatives, while superior to several of the synthetic and natural derivatives. The results show three fluoroquinolones to be potent helicase inhibitors that can be repurposed as supplemental therapy against HCV especially in cases non-responsive to DAAAs.

## Background:

Hepatitis C virus (HCV) is still a major public health issue in the 20th century, responsible for about 71 million infections worldwide [1]. Out of the 10 proteins encoded in the HCV genome [2], HCV non-structural protein 3 (NS3), which is a helicase belonging to RNA helicase super-family 2 enzymes, is one of the most crucial proteins [3]. The N-terminal domain of protein possesses serine protease activity in the presence of the NS4A cofactor protein, while the C-terminal exhibits helicase activity [4]. The N-terminal protease and C-terminal helicase domains of HCV NS3 are interdependent, and both enzymatic activities are essential for HCV replication, assembly, and pathogenesis [5]. Although current treatment therapy, comprising of directly acting antiviral agents (DAAA), is highly effective [6], still virus in 4-5% of the individuals do not respond to the therapy [7]. Furthermore, numerous reports have identified the emergence of drug resistance mutations, which can affect the activity of DAAAs [8]. These observations warrant the search for alternate or supplemental antiviral agents that can exhibit superior antiviral activity [9]. Several studies have reported the inhibitory activity of synthetic and natural compounds against HCV helicase [10]. Similarly, fluoroquinolones, which are broad-spectrum DNA gyrase and topoisomerase IV (helicase) enzyme inhibitors, have also been reported by us and others to exhibit inhibitory activity against the HCV helicases [11- 13]. Therefore, it is of interest to document the Molecular docking analysis of fluoroquinolones and other natural and synthetic compounds with the HCVNS3 helicase.

## Materials and Methods:

### HCV NS3 helicase structures:

The HCVNS3 helicase crystal structures (genotype 1a: PDB ID:1A1V and genotype 1b: PDB ID: 1CU1) wereretrieved from PDB database [14] in .pdb format (Figure 1 - see PDF).The structures wereedited to remove water molecules and any bound ligands in Discovery Studio Visualizer version 4.0 (DSV4.0; Dassault Systems BIOVIA, Discovery Studio Visualizer, version 4.0, San Diego: Dassault Systems, 2020) and thereafter saved in PDB format. The polar hydrogen and Kollman charges were added to the structures using Autodock tools [15], and structures were saved in .pdbqt format.

### HCV NS3 helicase inhibitors:

We compared the efficacy of 20 previously published synthetic and natural NS3 helicase inhibitors, namely, Ring-expanded (fat) nucleoside analogs [16], DRBT [17], manoalide [18], AICAR analog (compound 4) [19], thiazolpiperazinylderivatives, TBBT [17], cholesterol sulfate-1 [20], NS3 peptide (p14) [21], QU663 [22], compound 17, and tropolone derivatives [23] against selected fluoroquinolones, namely moxifloxacin, ofloxacin, and sparfloxacin that were previously reported to exhibit superior inhibitory activity against HCV NS3 helicase as compared to other fluoroquinolones tested [24].Structures of these drugs were retrieved from the PubChem database in SDF format [25] and converted into PDB format using OpenBabel software [26] (Figure 2 - see PDF).

### Molecular docking to analyze binding mode of inhibitors with HCV NS3:

For the analysis of drug-protein interactions, molecular docking simulations were performed. Molecular docking studies and conformational analysis were performed using a web-based version of Autodock Vina, Webina 1.0.3 [27], and interactions were analyzed in DSV 4.0 software. For docking studies, the X, Y, and Z grid box centers were set to 19 Å, 71Å, and 111 Å, respectively, while the X, Y, and Z box size-was set to 116 Å, 81 Å, and93 Åto cover the entire length of the protein. We adopted a blind docking approach to allow drugs to bind anywhere on the protein [28]. Molecular docking was performed using standard precision protocols with default parameters of Webina 1.0.3. Out of the stimulated interactions, the top 10 poses were selected based on docking energies, for these selected poses, further analysis of the interaction(s) between NS3-drug binding was carried out. Visualization of docking poses and analysis of drug-protein interactions were performed using DSV4.0.

## Results and Discussion:

The molecular docking analysis revealed that, in the case of NS3 helicase from genotype 1a, the fluoroquinolones; of loxacin, moxifloxacin, and sparfloxacin, as well as other NS3 inhibitors, formed interactions with the NS3 helicase in the catalytic groove (Figure 3 - see PDF). On the contrary, in the case of NS3 helicase from genotype 1b, the ligands formed interactions with NS3 helicase in three different regions, where sparfloxacin, thiazolpiperazinyl derivatives 3 and 5, and acridone derivative (Figure 3 - see PDF) bound in the catalytic domain of the helicase, while ofloxacin, TBBT, fatsol, and thiazolpiperazinyl derivatives 1, 2 and 4 (Figure 3 - see PDF) bound to the allosteric site of the helicase (Figure 3 - see PDF). Analysis of the docking energies revealed a strong binding affinity between NS3 helicase and the acridone derivatives (-11.4KJ/mol), compound 17 (-10.3KJ/mol), and thiazolpiperazinyl derivatives 1-5 (-9.7KJ/mol, -11.1KJ/mol, -10.6KJ/mol, -9.6KJ/mol, -9.8KJ/mol, respectively) (Figure 3 and Table 1 - see PDF). The fluoroquinolones namely, moxifloxacin (-9.1KJ/mol) and sparfloxacin (-9.2KJ/mol) also exhibited a high binding affinity with the NS3 helicase, comparable to thiazolpiperazinyl derivatives 1-5, and superior to several of the synthetic and natural derivatives (Figure 4 and Table 1 - see PDF). In the case of genotype 1b (1CU1), analysis of docking energies revealed a strong binding affinity between NS3 helicase and the acridone derivatives (-11.9KJ/mol), compound 17 (-10.8KJ/mol), and thiazolpiperazinyl derivatives 1-5 (-10.9KJ/mol, -10.9KJ/mol, -10.2KJ/mol, -9.6KJ/mol, -11.8KJ/mol respectively). Besides this, fluoroquinolones namely, ofloxacin (-9.2KJ/mol) and sparfloxacin (-9.2KJ/mol) also showed a higher affinity towards the catalytic region, which was superior to several of the synthetic and natural derivatives (Figure 5 and Table 1 - see PDF). Based on the binding energy values, the order of efficacy of interaction in between the fluoroquinolones and NS3 enzyme is in order, ofloxacin > moxifloxacin > sparfloxacin for genotype 1a. For genotype 1a, analysis of the drug-protein interactions revealed that amino acids, Glutamine-493, Proline-230, Aspartate-296, Threonine-295, Glutamate-460, and Glutamic acid-496 in the groove of the active site, were involved in the binding with all the three fluoroquinolones, whereas other synthetic and natural derivatives made strong hydrogen bonds with Valine-372, Lysine-373, and Tyrosine-350.These amino acids located within these catalytic regions are reported to be crucial for the enzyme activity and the HCV lifecycle, where Threonine-295 and Proline-230 mediate ATPase dependent binding to the active site, and aids in the maintenance of the electrostatic environment needed for ssDNA and RNA binding, thereby controlling the helicase activity [29,30]. In the case of genotype 1b, fluoroquinolones sparfloxacin and ofloxacin showed higher affinity towards the enzyme. The amino acids most commonly targeted by these fluoroquinolones includedThreonine-295 and Proline-230 Glutamine-493 having proximity toTryptophan-501. Glutamine-493 and Tyrosine-501,along with Proline-230 and Threonine-295 have a role in the ATPase-dependent binding of ssDNA and RNA within the DNA binding cleft, essential forhelicase activity [31]. Targeting these amino acids by ofloxacin and sparfloxacin in the catalytic region may result in the prevention of DNA binding and thereby ceasing the enzyme activity. The results for molecular docking are in agreement with another study reporting the in vitro inhibition of NS3 helicase, where the IC50 of ofloxacin was reported to be 0.1 µM, while the IC50 of moxifloxacin and sparfloxacin lied within the range of 0.1-10 µM [24], which was superior to some of the most potent NS3 helicase inhibitor tested, such as acridone derivatives, NS3 peptide (p14), QU663 and DRBT reportedly having IC50 values in the range of 10-20 µM [10], making fluoroquinolones better in-vitro candidates for the inhibition of NS3 activity.

## Conclusion:

Data shows fluoroquinolones that because of their established safety profile can be repurposed as a supplemental therapy against HCV, especially against the infections that arenon-responsive to DAAAs.
